# Season of birth and the risk of dementia in the population‐based Rotterdam Study

**DOI:** 10.1007/s10654-021-00755-3

**Published:** 2021-05-17

**Authors:** Sanne S. Mooldijk, Silvan Licher, Elisabeth J. Vinke, Meike W. Vernooij, Mohammad Kamran Ikram, Mohammad Arfan Ikram

**Affiliations:** 1 Department of Epidemiology, Erasmus MC University Medical Center, PO Box 2040, 3000 CA Rotterdam, The Netherlands; 2 Department of Radiology and Nuclear Medicine, Erasmus MC University Medical Center, Rotterdam, The Netherlands; 3 Department of Neurology, Erasmus MC University Medical Center, Rotterdam, The Netherlands

**Keywords:** Alzheimer’s disease, Dementia, Early life, Environmental factors, MRI

## Abstract

Early-life environmental factors have been suggested in the pathophysiology of dementia. Season of birth has previously been used as a proxy for these external exposures. We investigated the link between season of birth and the risk of dementia and further explored underlying pathways by studying structural brain changes on MRI. From the Dutch, population-based Rotterdam Study, 12,964 participants born between 1887 and 1960 were followed between 1990 and 2018 for dementia. Cox regression was conducted to assess the association between season of birth and dementia. In addition, we distinguished between mild and cold winters. The association of season of birth with structural brain markers on MRI was examined in 5237 participants. The risk of dementia in participants born in winter and fall was higher than of those born in summer (hazard ratio (HR) 1.15 [95% confidence interval (CI) 1.01–1.31] for winter and HR 1.17 [95% CI 1.01–1.33] for fall), especially for Alzheimer’s disease (HR 1.23 [1.06–1.43] for winter and HR 1.15 [95% CI 0.99–1.35] for fall). The risk was particularly increased for participants born in a cold winter. Except for slightly lower hippocampus in fall born participants (β − 0.03; 95% CI − 0.06 to 0.00), we did not find associations with brain imaging markers. In conclusion, winter and fall births were associated with a higher incidence of dementia, especially of AD. We did not find evidence for structural brain changes as an underlying mechanism.

**Supplementary Information:**

The online version contains supplementary material available at 10.1007/s10654-021-00755-3.

## Introduction

Dementia, including Alzheimer’s disease (AD), is a multifactorial disease with part of the individual predisposition shaped early in life [1–3]. These early-life influences are not only reflected by the substantial heritability of dementia, but increasingly environmental factors, such as cold temperatures, lack of sunlight and poor nutrition, have also been suggested as risk factors [4]. Impaired neurodevelopment leading to adverse outcomes later in life may mediate this and might be expressed in subtle differences in overall brain structure as well as in specific regions, such as the hippocampus [5, 6].

Season of birth has repeatedly been used as a proxy for the effect of several environmental exposures early in life on the risk of brain diseases later in life, including Parkinson’s disease, multiple sclerosis and schizophrenia [7–12]. Previous studies on season of birth and dementia generally show ambiguous results and are described in Table 1 [13–19, 4, 20, 21].

**Table 1:** Summary of previous studies on season of birth and dementia

Study	Location	Cases	Reference population	Result^a^	Conclusion
Philpot et al. [13]	United Kingdom	534 AD cases	Birth rates from age-matched census sample	75 versus 59.1	Excess of first-quarter births among persons with AD with no family history of dementia (47 vs. 29.4). Effect not found in persons with family history (12 vs. 18.4) or combined
Dysken et al. [14]	United States of America	727 Autopsy-confirmed AD cases	Birth rates from general population	202 versus 184	No differences between observed and expected quarterly birth rates, for both persons with and without family history
Vitiello et al. [15]	United States of America	150 AD cases	132 persons recruited from community	41 versus 28	No seasonal variation could be demonstrated. Did not change when excluding patients with family history of dementia
Henderson et al. [16]	Australia	170 AD cases	Birth rates from geographically close community sample of similar age-group	33 versus 38	No evidence for seasonality of birth was found
Fratiglioni et al. [17]	Sweden	121 AD cases	345 age and sex matched individuals from same cohort study, without dementia	1^st^ quarter RR = 1.4 (95% CI 0.9–2.3)^b^	First quarter of the year as season of birth was not associated with an increased risk of AD, even when only looking at cases without family history
Vézina et al. [18]	Canada	399 AD cases	(1) Population currently living in the area and (2) the population born during the same period in the area	May: 20 versus 33.8^c^	Significant deficit of births in the month of May
Ptok et al. [19]	Germany	131 AD cases	107 healthy elderly persons from same area	20 versus 19.4	Seasonal distribution of births was not found to increase the risk of AD. Controlling for *APOE* genotype did not change results
Doblhammer et al. [4]	Germany	14,718 dementia cases	149,225 persons (aged > 65) from the same health insurer	Winter: OR = 0.93^d^	Winter-born have the lowest risk of developing dementia. The highest risk was found among summer born. This difference was statistically significant when adjusting for age, sex and major vascular risk factors of dementia
Tolppanen et al. [20]	Finland	70,719 AD cases	282,862 age, sex and region-of-residence matched controls	Summer: OR = 1.03 (95% CI 1.00–1.05)^e^	No strong evidence that month or season of birth is related to risk of AD, although summer births (June–August) were associated with higher odds of AD compared to winter births. However, the absolute difference was only 0.5% (31.7% vs. 32.2%)
Ding et al. [21]	China	1326	354,859 respondents of the Second China National Sample Survey on Disability, aged 60 years and older	OR = 0.76 (95% CI 0.65–0.90)^f^	Winter birth was associated with a lower prevalence of dementia, especially among those living in urban and northern areas of China

*AD* Alzheimer’s disease, *CI* confidence interval, *OR* odds ratio, *RR* relative risk

^a^Results are shown for observed dementia births (cases with and without family history combined) versus expected dementia births (based on reference population) during the first quarter of the year, unless specified differently

^b^Risk ratio for AD comparing 1st quarter birth to birth during rest of the year, adjusted for age, sex and education

^c^Adjusted expected birth frequency based on population currently living in the area

^d^Odds ratio for dementia among winter born (Dec.–Feb.) compared to summer born (Jun.–Aug.), adjusted for age, sex and major vascular risk factors of dementia

^e^Odds ratio for dementia among summer born (Jun.–Aug.) compared to winter born (Nov.–Feb.)

^f^Odds ratio for dementia among summer born (Jun.–Aug.) compared to winter born (Dec.–Feb.), adjusted for age, gender, education, and annual household income

In addition to season of birth, another way to summarize environmental exposures early in life is to use Hellmann scores, reflecting the annual severity of the winters in the Netherlands. A comparison of dementia incidence across birth years could further inform on the link between cold temperatures in early life and dementia [22].

We investigated the association of early life environment, using both season of birth and severity of winters as reflected by the Hellmann scores, with the risk of dementia. We further sought to identify the underlying pathological substrate by studying volumetric and microstructural brain markers on MRI.

## Methods

### Setting and study population

This study was conducted within the Rotterdam Study, a prospective population-based cohort study, that started in 1990 with 7983 participants aged 55 years and over. In 2000, 3011 participants were added as a second recruitment wave and in 2006, 3932 persons aged 45 years and over were added as a third recruitment wave. Follow-up examinations at the research center take place every 4–6 years. In addition, all participants were continuously monitored through electronic linkage of the study database with their medical records. Details of the study have been previously described elsewhere [23]. For the current study, we selected all participants that attended the research center at least once for examination. Participants who did not visit the research center were excluded from the analyses (n = 1397), because inclusion would lead to excessive missing data as most of the selected covariates were measured at the research center. Of the remaining participants, 73 had insufficient screening for dementia and 394 had a history of dementia at study entry. Of the 13,062 participants without dementia at baseline, 98 did not provide informed consent for dementia follow-up and were excluded. The remaining 12,964 participants were included in the analyses of risk of dementia. From 2005 onwards, brain MRI was implemented in the Rotterdam Study protocol. For the present study, we used the first available scan for each participant without dementia, Parkinson or stroke at the moment of scanning. In addition, all scans with MRI-defined cortical infarcts were excluded from the analyses (n = 132 scans), leaving scans of 5237 persons for analyses.

The Medical ethics committee at the Erasmus University of Rotterdam and the Ministry of Health, Welfare and Sport of the Netherlands approved the Rotterdam Study. The study is implemented in the “Wet bevolkingsonderzoek: ERGO (Population Studies Act: Rotterdam Study)”. All participants provided written informed consent to participate in the study and for the researchers to obtain information from their treating physicians.

### Season of birth

We used the meteorological distribution of the seasons which defines winter as December 1st to February 28th/29th, spring as March 1st to May 31st, summer as June 1st to August 31st and fall as September 1st to November 30th [24].

### Severity of winters

To differentiate between being born in cold and in normal winters, we used Hellmann numbers as an indicator. This number is calculated annually since 1900 in the Netherlands as the average temperature per day from November 1st of the previous year to March 31st of the corresponding year and summing the number of days with average below 0 °C [22]. Hellmann numbers reflect the severity of a winter and can be categorized into mild winters (Hellman score ≤ 100), cold winters (100–160) and very cold winters (> 160).

### Assessment of dementia

The Mini-Mental State Examination and the Geriatric Mental Schedule organic level were used to screen for dementia at baseline and at subsequent center visits [25]. Cut offs were < 26 for the Mini-Mental State Examination and > 0 for the Geriatric Mental Schedule. Participants with a positive screening outcome underwent further testing using the Cambridge Examination for Mental Disorders of the Elderly [25]. Additionally, the electronic linkage of the medical records from general practitioners and the regional institute for outpatient mental health care was used for the dementia diagnosis. Available information on cognitive testing and clinical neuroimaging was used when required for diagnosis of dementia subtype. The final diagnosis was established by a consensus panel led by a consultant neurologist, according to standard criteria for dementia (DSM-III-R) and AD (National Institute of Neurological and Communicative Disorders and Stroke-Alzheimer’s Disease and Related Disorders Association (NINCDS-ADRDA)).

### Brain imaging

Brain MRI scanning was done in the Rotterdam Study population from 2005 onwards, using a 1.5-Tesla MRI scanner (GE Signa Excite; GE Healthcare, Milwaukee, USA) to obtain structural markers (intracranial volume, total brain volume, gray matter volume, white matter volume, white matter hyperintensity volume and hippocampus volume), and white matter microstructural integrity markers (fractional anisotropy and mean diffusivity). The scan protocol, sequence details and processing of MRI data in the Rotterdam study were previously described elsewhere [23, 26].

### Assessment of covariates

Measured covariates were ethnicity, educational level, income, apolipoprotein E (APOE) genotype, cardiovascular risk factors, including body mass index, smoking and history of cardiovascular diseases, including myocardial infarction and revascularization procedures, diabetes, stroke and depressive symptoms.

Information about ethnicity, educational level, income, alcohol consumption and smoking habits was obtained by trained interviewers during home interviews [23]. Educational level was categorized into four groups, ranging from primary to high education (university). Alcohol use was categorized into abstainers and users. Smoking behaviour was categorized as never smoker, former smoker, and current smoker.

Body mass index was calculated as weight in kilograms divided by squared height in meters. Blood pressure was measured in a sitting position on the right arm using a random-zero sphygmomanometer. The average of two measurements was used for analysis. Total cholesterol and high-density lipoprotein cholesterol were acquired by an automated enzymatic procedure (Boehringer Mannheim System). *APOE* genotype was determined by polymerase chain reaction in the original cohort and by bi-allelic TaqMan assay in the extended cohorts on coded DNA samples without knowledge of the dementia diagnosis [27, 28]. *APOE* genotype was categorized for analysis as no ε4 allele, carrier of one ε4 allele or carrier of two ε4 alleles.

Diabetes mellitus was diagnosed as fasting blood glucose ≥ 7.0 mmol/L or use of anti-diabetic drugs obtained by interview and pharmacy records [29]. History of stroke, coronary heart disease, and heart failure status was assessed by self-report and verified by continuous monitoring of medical records through digitized linkage of files from general practitioners with the study database [30]. Depressive symptoms were assessed by using the validated Center for Epidemiology Depression Scale with a score of 16 or higher considered suggestive of depressive symptoms [31].

### Statistical analysis

The association of season of birth with dementia was assessed in survival analyses, using Cox proportional hazard models. Initially, we adjusted for age and sex (Model 1). We additionally adjusted for educational level in Model 2 to explore its possible role as a mediator. This is related to the situation in the Netherlands, in which children with a birthdate after the summer holidays (i.e. September and later) usually have to wait until the following summer to enrol in primary school. These children will thus be the oldest in their class, potentially giving them an advantage in educational performance, which in turn is a protective factor against dementia. In a third model (Model 3), we additionally adjusted for ethnicity, income, use of alcohol, smoking, body mass index, blood pressure, total and high-density lipoprotein cholesterol, *APOE* genotype, history of diabetes, history of stroke, history of coronary heart disease, history of heart failure and depressive symptoms. Follow-up time was defined as the time between the first center visit until date of dementia diagnosis, date of death, loss to follow-up, or the administrative end of study (January 1st, 2018), whichever came first. Follow-up until January 1st, 2018 was near complete (96 % of potential person-years).

We expected summer born participants to have the lowest risk of dementia, as they were less likely to be exposed to extreme temperature, viral infections and nutritional deficiencies that may damage the central nervous system, as is suggested to have a role in seasonality of birth in schizophrenia [32]. In line with that, we chose this group as the reference for the analyses. We studied associations for AD separately and we studied associations stratified by *APOE* ε4 carriership. In addition, we repeated the main analysis with month of birth as the determinant with July as the reference.

To compare individuals born in mild winters (Hellmann score ≤ 100) versus cold (> 100) or very cold (> 160) winters, we used the previously mentioned models for adjustment, with summer birth as the reference. For this analysis, we excluded participants born before 1900 (N = 55) because Hellmann scores were not available for those years.For the second aim of our study we studied the association between season of birth and brain imaging markers. To this end, we used linear regression with adjustment for age, sex and intracranial volume. We also investigated whether adjustments for age-squared would give a better adjustment for confounding by age. Moreover, we tested whether the results were driven by participants with prodromal dementia by performing the analysis after excluding participants who developed dementia before January 1st, 2018.

Missing data on covariates were imputed using 5-fold multiple imputation. Data were missing for ethnicity (4.6 %), educational level (1.8 %), income (12.9 %), alcohol use (10.8 %), smoking (1.4 %), body mass index (2.0 %), systolic blood pressure (1.4 %), diastolic blood pressure (1.4 %), total cholesterol (2.5 %), high-density lipoprotein cholesterol (2.6 %), *APOE*-ε4 carrier status (6.0 %), history of diabetes (9.1 %), history of coronary heart disease (3.2 %), history of heart failure (0.2 %) and depressive symptoms (20.5 %). We performed a complete-case analysis to determine the effect of the imputations on our results. All analyses were conducted using R version 3.6.3. We considered results statistically significant when the *P* value was below 0.05.

## Results

Baseline characteristics of the study population used in the dementia analyses (n = 12,964) are presented in Table 2. The characteristics were largely similar across seasons of birth (Supplementary Table 1).

**Table 2:** Population characteristics of participants in the Rotterdam study (1990–2016)

Characteristic	All participants (N = 12,964)		Participants with MRI (N = 5237)	
	Mean (SD)	No. (%)	Mean (SD)	No. (%)
Age, years	64.8 (9.6)		64.3 (10.6)	
Women		7486 (57.7)		2939 (56.1)
Caucasian descent		11,946 (96.5)		4822 (94.5)
Educational level				
Primary		2115 (16.6)		456 (8.8)
Lower		5155 (40.5)		1976 (38.2)
Intermediate		3531 (27.7)		1546 (29.9)
Higher		1936 (15.2)		1195 (23.1)
Income, euros/year				
< 25,000		2240 (19.8)		352 (7.5)
25,000–45,000		4225 (37.4)		1356 (28.7)
45,000–65,000		2477 (21.9)		1337 (28.3)
> 65,000		2352 (20.8)		1679 (35.5)
Alcohol use		8991 (84.3)		4583 (88.0)
Smoking				
Never		4124 (32.3)		1722 (33.1)
Former		5581 (43.7)		2617 (50.3)
Current		3072 (24.0)		866 (16.6)
Body mass index, kg/m^2^	26.9 (4.1)		27.5 (4.2)	
Systolic blood pressure, mmHg	138.3 (21.7)		139.5 (21.3)	
Diastolic blood pressure, mmHg	77.4 (11.9)		82.6 (10.9)	
Total cholesterol, mmol/L	6.16 (1.23)		5.56 (1.05)	
High-density lipoprotein cholesterol, mmol/L	1.37 (0.39)		1.45 (0.42)	
*APOE*-ε4 carrier status				
Non-carrier		8761 (71.9)		3507 (71.9)
1 allele		3154 (25.9)		1265 (25.9)
2 alleles		276 (2.3)		104 (2.1)
History of diabetes		1517 (12.9)		580 (11.6)
History of coronary heart disease	335 (2.6)		191 (3.7)	
History of heart failure	825 (6.6)		21 (0.4)	
History of stroke	272 (2.1)		0 (0.0)	
Depressive symptoms	899 (8.7)		411 (8.1)	

*APOE* apolipoprotein E, *SD* standard deviation

**Fig. 1:**
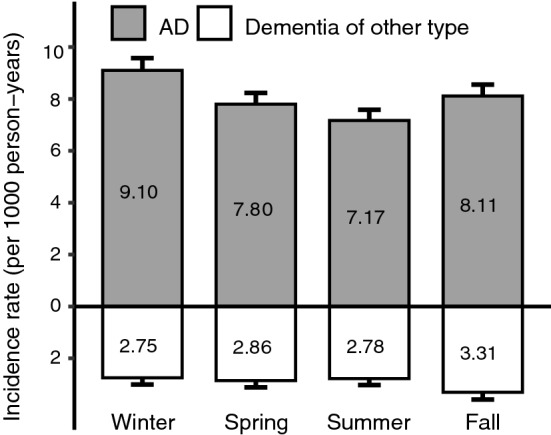
Incidence rate of dementia by season of birth. Incidence rates of dementia per 1000 person-years of follow-up by season of birth. Gray bars represent the incidence rate of Alzheimer’s disease. White bars represent other forms of dementia. Error-bars represent the standard error for the group. *AD* Alzheimer’s disease

During 168,867 person-years of follow-up (median 11.4 years), 1,850 participants developed dementia (median age 83.6; 67.9 % women), of whom 1357 had AD. The incidence rate of dementia per 1000 person-years ranged from 9.9 (95% CI 9.0–10.9) among summer born participants to 11.9 (95% CI 10.8–12.9) among winter born participants (Fig. 1). The lowest incidence rate was seen in participants born in July and the highest in participants born in February (9.2 (95% CI 7.7–10.8) and 12.4 (95% CI 10.5–14.2), respectively; Supplementary Table 2).

Adjusted for age and sex (Model 1), the risk of developing dementia was increased among participants born in winter, spring and fall compared to summer born participants (hazard ratio (HR) 1.15 (95% CI 1.01–1.31) for winter, HR 1.12 (95% CI 0.98–1.28) for spring and HR 1.17 (95% CI 1.03–1.33) for fall (Table 3)). The results by month of birth are shown in Supplementary Table 2.

**Table 3:** Associations of season of birth with risk of dementia and of Alzheimer’s disease, Rotterdam study, the Netherlands, 1990–2018

Season of birth	n/N	Model 1		Model 2		Model 3	
		HR	95% CI	HR	95% CI	HR	95% CI
*All-cause dementia*							
Winter (Dec., Jan., Feb.)	491/3204	1.15	1.01–1.31	1.16	1.02–1.32	1.13	0.99–1.29
Spring (Mar., Apr., May.)	458/3330	1.12	0.98–1.28	1.12	0.98–1.28	1.10	0.96–1.26
Summer (Jun., Jul., Aug.)	428/3256	1.00	Referent	1.00	Referent	1.00	Referent
Fall (Sep., Oct., Nov.)	473/3174	1.17	1.03–1.33	1.17	1.03–1.33	1.10	0.96–1.26
*Alzheimer’s disease*							
Winter (Dec., Jan., Feb.)	377/3204	1.23	1.06–1.43	1.23	1.06–1.43	1.20	1.03–1.40
Spring (Mar., Apr., May.)	335/3330	1.14	0.98–1.33	1.14	0.98–1.33	1.13	0.96–1.32
Summer (Jun., Jul., Aug.)	309/3256	1.00	Referent	1.00	Referent	1.00	Referent
Fall (Sep., Oct., Nov.)	336/3174	1.15	0.99–1.35	1.15	0.99–1.35	1.08	0.92–1.26

*CI* confidence interval, *HR* hazard ratio, *n* number of dementia cases, *N* total sample size

Model 1 is adjusted for age and sex. Model 2 is additionally adjusted for education. Model 3 is additionally adjusted for ethnicity, body mass index, systolic blood pressure, diastolic blood pressure, smoking, history of diabetes mellitus, alcohol use, total cholesterol, high-density lipoprotein cholesterol, *APOE* ε4 genotype, history of heart failure, history of coronary heart disease, history of stroke and depressive symptoms

The same pattern with somewhat stronger associations was found for AD (HR 1.23 (95% CI-1.06, 1.43) for winter, HR 1.14 (95% CI 0.98–1.33) for spring, and HR 1.15 (95% CI 0.99–1.35) for fall.

Similar patterns were found when additionally adjusting for educational level (Model 2), and for ethnicity, income, *APOE ε4* genotype, cardiovascular risk factors and history of depressive symptoms (Model 3) (Table 3). Using Model 3, the risk of both all-cause dementia and of AD was highest among winter born participants (HR 1.13 (95% CI 0.99–1.29) for all-cause dementia and HR 1.20 (95% CI 1.03–1.40) for AD).

In the analyses stratified by *APOE* ε4 carrier ship, similar patterns were found for carriers (n = 3430) and non-carriers (n = 8761) (Supplementary Table 3). The complete-case analysis did not substantially change the results (Supplementary Table 4).

### Winter coldness

The risk for developing dementia was particularly increased in individuals born in a cold winter (Hellman score 100–160, n = 405) or very cold winter (Hellmann score > 160, n = 393) compared to those born in summer, with age and sex adjusted HR 1.34 (95% CI 1.05–1.71) and 1.35 (95% CI 0.99–1.84), respectively. This pattern remained when using Model 3 for adjustment (Table 4). Hellmann scores by birth year are shown in Supplementary Fig. 1.

**Table 4:** Associations of winter severity at birth with incident dementia, Rotterdam study, the Netherlands, 1990–2018

Winter severity	n/N	Model 1		Model 2		Model 3	
		HR	95% CI	HR	95% CI	HR	95% CI
Normal winter (H ≤ 100)	362/2386	1.10	0.95–1.26	1.10	0.96–1.27	1.10	0.95–1.26
Cold winter (H 100–160)	79/405	1.34	1.05–1.71	1.35	1.06–1.72	1.29	1.01–1.65
Very cold winter (H > 160)	44/393	1.35	0.99–1.84	1.35	0.99–1.85	1.34	0.98–1.83

Participants born in normal, cold and very cold winters, based on Hellmann scores (≤ 100, 100–160 and < 160, respectively) are compared to participants born in summer

CI, confidence interval; H, Hellmann score; HR, hazard ratio; n, number of dementia cases; N, total sample size

Model 1 is adjusted for age and sex. Model 2 is additionally adjusted for education. Model 3 is additionally adjusted for ethnicity, body mass index, systolic blood pressure, diastolic blood pressure, smoking, history of diabetes mellitus, alcohol use, total cholesterol, high-density lipoprotein cholesterol, *APOE* ε4 genotype, history of heart failure, history of coronary heart disease, history of stroke and depressive symptoms

### Brain imaging markers

Among 5237 dementia-free participants with a brain MRI scan available (mean age 64.3 (SD 10.6), 56.1 % females, Table 2), we did not find differences in structural and microstructural brain markers between participants born in different seasons, except for hippocampus volume, with a lower volume in fall-born participants compared to the summer born participants (adjusted difference − 0.03 (95% CI − 0.06, 0.00), *P* = 0.03). Results for total brain volumetrics are shown in Table 5 (further results in Supplementary Table 5). These results did not change after additionally adjusting for age squared and after excluding participants with prodromal dementia (data not shown).

**Table 5:** Associations of season of birth with brain imaging markers, Rotterdam study, the Netherlands, 1990–2018

Season of birth	Total brain volume		Gray matter volume		White matter volume		Hippocampus volume	
	β	95% CI	β	95% CI	β	95% CI	β	95% CI
Winter (Dec., Jan., Feb.)	− 0.09	− 2.55, 2.38	− 0.42	− 3.21, 1.34	0.85	− 1.80, 3.50	− 0.01	− 0.05, 0.02
Spring (Mar., Apr., May.)	0.51	− 1.90, 2.92	0.69	− 1.77, 2.69	0.05	− 2.54, 2.65	− 0.01	− 0.04, 0.02
Summer (Jun., Jul., Aug.)	Referent	Referent	Referent	Referent				
Fall (Sep., Oct., Nov.)	− 1.32	− 3.78, 1.14	0.46	− 1.41, 3.14	− 2.18	− 4.83, 0.47	− 0.03	− 0.06, 0.00

*CI* confidence interval

Differences in brain volumes (mL) with adjustment for age, sex and intracranial volume

## Discussion

In this population-based study in the Netherlands, winter and fall births were associated with a higher incidence of dementia, especially of AD. Particularly those born in a cold winter were at increased risk compared to those born in a normal winter. We did not find an association between season of birth and structural brain imaging markers later in life, except for a lower hippocampal volume for fall-born participants compared to summer-born participants.

So far, studies that assessed the relationship between environmental exposures early in life and dementia or AD, using season of birth as a proxy, showed inconsistent results [13–19, 4, 20, 21]. Small sample size and a discrepancy between the population from which the cases were selected and the reference population, may have led to these varying results. This prospective population-based study aimed to clarify this ambiguity, and its closed cohort design enabled us to study the association of season of birth with dementia in a large cohort in which people that developed dementia compared to those who did not arose from the same population.

Our findings may be explained by a seasonal distribution of harmful circumstances. For example, infants born in winter are more likely to be exposed to extreme temperature [33], nutritional or vitamin deficiencies [34, 35] and infections. This may affect the risk of poor cognitive functioning and dementia later in life, via damage or poor development of the central nervous system [36–38], metabolic adaptations and activation of inflammatory pathways during mid-life [4, 39, 40]. We further supported this by showing a slightly higher incidence of dementia among participants born in harsh winters.

Finally, educational level is suggested to convey the effect of season of birth on diseases later in life [4]. In the Netherlands, children with a birthdate after a certain deadline for school attendance have to wait a full school cycle, i.e. up to 10 months, until they can enrol in school. These children will be the oldest in their class, potentially giving them an advantage over their classmates. However, we did not find this advantage for fall born individuals. Furthermore, in a model that included educational level to test this potential mediating pathway, we found nearly identical results.

The stronger association for AD indicates that environmental factors around the moment of birth may affect the risk of AD to a greater extent than of other dementia types. However, since AD was clinically diagnosed and was not confirmed by neuropathological data, we were not able to draw conclusions on whether the early-life environment affects the amyloid and tau burden.

It is important to acknowledge the potential influence of historical events that may affect birth rates, child mortality and the incidence of diseases later in life. In this population in particular, the second world war and the Dutch famine of 1944–1945 in which there were widespread food shortages in the Netherlands, are likely to influence such outcomes and may bias the associations of season of birth with diseases later in life [41, 42]. In the present study, few participants that developed dementia during follow-up were born in this period (Supplementary Fig. 2) which makes it unlikely that the overall results are driven by this group.

A limitation of this study is that our findings may not be generalizable to other places and populations, as the associations probably depend on seasonal circumstances such as weather and latitude. Secondly, the Hellmann number is a summary measure for coldness over a five-month period and may not always reflect the coldness that someone endured during early life. For example, misclassification may occur for individuals born at the end of March as they endured only a small part of this 5-month period, potentially leading to a dilution of the found association.

Thirdly, the MRI scans were made at later age and although we selected the first scan for each participant, early dementia-related pathology may already have been present. However, the results did not change when participants with dementia in the near future were excluded.

A more general consideration is that the presumed underlying seasonal factor remains more or less unknown. For example, it may not be the moment of birth that results in a higher risk of dementia, as the association could also be explained by circumstances at the moment of conception, during the first, second or third trimester, or after pregnancy. Further knowledge about the most critical moments or periods are needed to draw any conclusions about causality and biological pathways in this field.

A strength of this study is the large sample size of our cohort, which is needed to find subtle differences in risk. Further strengths are the near-complete dementia follow-up, reducing the possibility of selective drop-out, and the meticulous ascertainment of dementia and covariates, which among others enabled to explore potential mediation by educational level.

In this western European population-based study winter births are associated with a higher incidence of dementia, especially of AD. This confirms the importance of early-life exposure in the pathophysiology of dementia and implicates affected neurodevelopment as a potential underlying mechanism. Since evidence for this mechanism was not found when focusing on structural and microstructural brain imaging markers, future studies are warranted to explore other biological mechanisms that may underlie this association, such as epigenetic marks, metabolic adaptations, intestinal microbiota and the immune system.

## Supplementary Information

Below is the link to the electronic supplementary material.

Supplementary file1 (DOCX 73 kb)

## Data Availability

Data can be obtained on request. Requests should be directed to the management team of the Rotterdam Study (secretariat.epi@erasmusmc.nl), which has a protocol for approving data requests. Because of restrictions based on privacy regulations and informed consent of the participants, data cannot be made freely available in a public repository.
