# Supplementary material for: Season of birth and the risk of dementia in the population‐based Rotterdam Study
Source: Eur J Epidemiol. 2021 May 17;36(5):497–506. doi: 10.1007/s10654-021-00755-3 (PMC8159812; doi:10.1007/s10654-021-00755-3)
Supplement: Supplementary file 1 — Supplementary file1 (DOCX 73 kb) [file 10654_2021_755_MOESM1_ESM.docx]

Season of birth and the risk of dementia in the population-based Rotterdam Study

Sanne S. Mooldijk, BSc^a^, Silvan Licher, MD^a^, Elisabeth J. Vinke, MSc^a,b^, Meike W. Vernooij, MD, PhD^a,b^, M. Kamran Ikram, MD, PhD^a,c^, M. Arfan Ikram, MD, PhD^a^*

^a^ Department of Epidemiology, Erasmus MC University Medical Center, Rotterdam, the Netherlands

^b^ Department of Radiology and Nuclear Medicine, Erasmus MC University Medical Center, Rotterdam, the Netherlands

^c^ Department of Neurology, Erasmus MC University Medical Center, Rotterdam, The Netherlands.

*Address correspondence to: M. Arfan Ikram, MD, PhD, Department of Epidemiology, Erasmus Medical Center, PO Box 2040, 3000 CA, Rotterdam, The Netherlands. E-mail: [m.a.ikram@erasmusmc.nl](mailto:m.a.ikram@erasmusmc.nl).

**Supplementary Material**

[**Supplementary Table 1** Baseline characteristics of the study population by season of birth 2](#_Toc65753747)

[**Supplementary Table 2** Associations of month of birth with the risk of dementia 4](#_Toc65753748)

[**Supplementary Table 3** Season of birth and incident dementia all-cause dementia and Alzheimer’s disease by *APOE* ε4 carriership 5](#_Toc65753749)

[**Supplementary Table 4** Associations of season of birth with the risk of dementia and of Alzheimer’s Disease in participants without missing covariates 6](#_Toc65753750)

[**Supplementary Fig. 1** Hellmann scores by birth year 7](#_Toc65753751)

[**Supplementary Table 4** Brain imaging and season of birth 8](#_Toc65753752)

[**Supplementary Fig. 2** Number of participants by year of birth 9](#_Toc65753753)

# Supplementary Table 1 Baseline characteristics of the study population by season of birth

|  |  | **Winter**:  Dec., Jan., Feb. | **Spring**:  Mar., Apr., May. | **Summer**:  Jun., Jul., Aug. | **Fall**:  Sep., Oct., Nov. | **P value** |
| --- | --- | --- | --- | --- | --- | --- |
| N | | 3,204 | 3,330 | 3,256 | 3,174 |  |
| Age, years | | 65.14 (9.74) | 64.67 (9.48) | 64.55 (9.51) | 64.69 (9.61) | 0.07 |
| Women | | 1,870 (58.4) | 1,905 (57.2) | 1,876 (57.6) | 1,835 (57.8) | 0.82 |
| Caucasian descent | | 2,939 (96.3) | 3,067 (96.4) | 3,020 (96.8) | 2,920 (96.7) | 0.64 |
| Educational level | |  |  |  |  | 0.48 |
| Primary | | 526 (16.7) | 547 (16.7) | 522 (16.3) | 520 (16.7) |  |
| Lower | | 1,289 (41.0) | 1,268 (38.8) | 1,325 (41.3) | 1,273 (40.9) |  |
| Intermediate | | 847 (26.9) | 952 (29.1) | 864 (26.9) | 868 (27.9) |  |
| Higher | | 485 (15.4) | 502 (15.4) | 497 (15.5) | 452 (14.5) |  |
| Income, euros/year | |  |  |  |  | 0.63 |
| <25000 | | 564 (20.3) | 570 (19.6) | 563 (19.8) | 543 (19.7) |  |
| 25000-45000 | | 1,065 (38.2) | 1,058 (36.4) | 1,041 (36.6) | 1,061 (38.4) |  |
| 45000-65000 | | 591 (21.2) | 638 (22.0) | 645 (22.7) | 603 (21.8) |  |
| >65000 | | 565 (20.3) | 638 (22.0) | 593 (20.9) | 556 (20.1) |  |
| Alcohol use | | 2,383 (83.5) | 2,490 (84.0) | 2,437 (83.1) | 2,345 (83.5) | 0.99 |
| Smoking | |  |  |  |  | 0.60 |
| Never | | 1,031 (32.7) | 1,026 (31.3) | 1,042 (32.4) | 1,025 (32.8) |  |
| Former | | 1,392 (44.1) | 1,464 (44.6) | 1,384 (43.0) | 1,341 (42.9) |  |
| Current | | 731 (23.2) | 789 (24.1) | 794 (24.7) | 758 (24.3) |  |
| Body mass index, kg/m^2^ | | 26.8 (4.0) | 26.9 (4.1) | 27.0 (4.1) | 27.0 (4.3) | 0.07 |
| Systolic blood pressure, mmHg | | 138.6 (22.1) | 138.8 (21.5) | 137.8 (21.2) | 138.0 (21.9) | 0.20 |
| Diastolic blood pressure, mmHg | | 77.1 (11.8) | 77.7 (12.0) | 77.4 (11.9) | 77.2 (11.9) | 0.34 |
| Total cholesterol, mmol/L | | 6.17 (1.23) | 6.15 (1.22) | 6.15 (1.23) | 6.17 (1.26) | 0.88 |
| High-density lipoprotein cholesterol, mmol/L | | 1.39 (0.40) | 1.37 (0.38) | 1.37 (0.40) | 1.37 (0.39) | 0.25 |
| *APOE*-ε4 carrier status | |  |  |  |  | 0.19 |
| Non-carrier | | 2,156 (71.1) | 2,275 (72.3) | 2,232 (73.3) | 2,098 (70.8) |  |
| 1 allele | | 813 (26.8) | 800 (25.4) | 754 (24.7) | 787 (26.5) |  |
| 2 alleles | | 63 (2.1) | 72 (2.3) | 61 (2.0) | 80 (2.7) |  |
| History of diabetes | | 371 (12.7) | 400 (13.2) | 383 (13.0) | 363 (12.5) | 0.90 |
| History of stroke | | 75 (2.3) | 91 (2.7) | 75 (2.3) | 94 (3.0) | 0.28 |
| History of coronary heart disease | | 195 (6.3) | 225 (7.0) | 209 (6.6) | 196 (6.4) | 0.69 |
| History of heart failure | | 75 (2.3) | 71 (2.1) | 69 (2.1) | 57 (1.8) | 0.50 |
| Depressive symptoms | | 191 (7.6) | 225 (8.5) | 252 (9.8) | 231 (9.1) | 0.04 |
| Dementia during follow-up | | 491 (15.3) | 458 (13.8) | 428 (13.1) | 473 (14.9) | 0.05 |
| Age at dementia diagnosis | | 83.7 (6.8) | 83.6 (6.8) | 83.5 (6.8) | 82.8 (7.1) | 0.14 |

Data are shown for non-imputed data of the study population (N=12,964). Values are counts (%) or means (standard deviation).

# Supplementary Table 2 Associations of month of birth with the risk of dementia

| **Month of birth** | **n/N** | **IR^a^** | **95% CI** | **Model 1^b^** | | **Model 2^b^** | | **Model 3^b^** | |
| --- | --- | --- | --- | --- | --- | --- | --- | --- | --- |
|  |  |  |  | **HR** | **95% CI** | **HR** | **95% CI** | **HR** | **95% CI** |
| January | 157/1097 | 11.2 | 9.4-12.9 | 1.24 | 0.98-1.56 | 1.24 | 0.98-1.56 | 1.22 | 0.97-1.55 |
| February | 168/1053 | 12.4 | 10.5-14.2 | 1.33 | 1.06-1.67 | 1.33 | 1.06-1.68 | 1.28 | 1.02-1.61 |
| March | 159/1123 | 10.9 | 9.2-12.6 | 1.27 | 1.01-1.60 | 1.27 | 1.01-1.60 | 1.22 | 0.97-1.54 |
| April | 143/1091 | 10.1 | 8.5-11.8 | 1.17 | 0.92-1.48 | 1.17 | 0.92-1.48 | 1.14 | 0.90-1.45 |
| May | 156/1116 | 10.9 | 9.2-12.7 | 1.27 | 1.01-1.60 | 1.28 | 1.02-1.62 | 1.28 | 1.01-1.61 |
| June | 145/1034 | 10.8 | 9.0-12.6 | 1.20 | 0.95-1.52 | 1.21 | 0.95-1.53 | 1.20 | 0.95-1.52 |
| July | 133/1099 | 9.2 | 7.7-10.8 | 1.00 | Referent | 1.00 | Referent | 1.00 | Referent |
| August | 150/1123 | 9.8 | 8.2-11.4 | 1.11 | 0.88-1.41 | 1.12 | 0.88-1.41 | 1.12 | 0.88-1.41 |
| September | 159/1105 | 11.3 | 9.5-13.0 | 1.26 | 1.00-1.59 | 1.27 | 1.01-1.60 | 1.21 | 0.96-1.53 |
| October | 162/1057 | 11.8 | 10.0-13.6 | 1.32 | 1.05-1.67 | 1.32 | 1.05-1.66 | 1.23 | 0.97-1.55 |
| November | 152/1012 | 11.2 | 9.4-12.9 | 1.28 | 1.01-1.62 | 1.29 | 1.02-1.63 | 1.20 | 0.95-1.52 |
| December | 166/1054 | 12.1 | 10.2-13.9 | 1.25 | 0.99-1.57 | 1.26 | 1.00-1.59 | 1.23 | 0.98-1.56 |

CI, confidence interval; HR, hazard ratio; IR, incidence rate; n, number of dementia cases; N, total sample size.
^a^ Incidence rates are calculated as events per 1,000 person-years of follow-up time.
^b^ Model 1 is adjusted for age and sex. Model 2 is additionally adjusted for education. Model 3 is additionally adjusted for ethnicity, body mass index, systolic blood pressure, diastolic blood pressure, smoking, history of diabetes mellitus, alcohol use, total cholesterol, high-density lipoprotein cholesterol, *APOE* ε4 genotype, history of heart failure, history of coronary heart disease, history of stroke and depressive symptoms.

# Supplementary Table 3 Season of birth and incident dementia all-cause dementia and Alzheimer’s disease by *APOE* ε4 carriership

| **Season of birth** | **All-cause dementia** | | | | |
| --- | --- | --- | --- | --- | --- |
|  | ***APOE* ε4 carriers** | |  | ***APOE* ε4 non-carriers** | |
|  | **n/N** | **HR (95% CI)** |  | **n/N** | **HR (95% CI)** |
| Winter (Dec., Jan., Feb.) | 188/876 | 1.15 (0.97-1.37) |  | 281/2,156 | 1.13 (0.91-1.40) |
| Spring (Mar., Apr., May.) | 175/872 | 1.12 (0.94-1.33) |  | 261/2,275 | 1.11 (0.89-1.38) |
| Summer (Jun., Jul., Aug.) | 151/815 | 1.00 Referent |  | 250/2,232 | 1.00 Referent |
| Fall (Sep., Oct., Nov.) | 197/867 | 1.15 (0.97-1.37) |  | 257/2,098 | 1.18 (0.95-1.46) |
|  | **Alzheimer’s disease** | | | | |
|  | ***APOE* ε4 carriers** | |  | ***APOE* ε4 non-carriers** | |
|  | **n/N** | **HR (95% CI)** |  | **n/N** | **HR (95% CI)** |
| Winter (Dec., Jan., Feb.) | 143/876 | 1.26 (1.03-1.54) |  | 219/2,156 | 1.13 (0.88-1.44) |
| Spring (Mar., Apr., May.) | 141/872 | 1.06 (0.86-1.31) |  | 175/2,275 | 1.17 (0.92-1.50) |
| Summer (Jun., Jul., Aug.) | 116/815 | 1.00 Referent |  | 176/2,232 | 1.00 Referent |
| Fall (Sep., Oct., Nov.) | 145/867 | 1.11 (0.90-1.37) |  | 175/2,098 | 1.14 (0.89-1.45) |

CI, confidence interval; HR, hazard ratio; n, number of dementia cases; N, total sample size.
Estimates are adjusted for age and sex (Model 1).

# Supplementary Table 4 Associations of season of birth with the risk of dementia and of Alzheimer’s Disease in participants without missing covariates

| **Season of birth** | **All-cause dementia** | | | | | |
| --- | --- | --- | --- | --- | --- | --- |
|  | **Model 2^a^** | | | **Model 3^a^** | | |
|  | **n/N** | **HR** | **95% CI** | **n/N** | **HR** | **95% CI** |
| Winter (Dec., Jan., Feb.) | 477/3,147 | 1.16 | 1.01-1.32 | 234/1,888 | 1.12 | 0.93-1.36 |
| Spring (Mar., Apr., May.) | 441/3,269 | 1.11 | 0.97-1.27 | 224/2,007 | 1.06 | 0.87-1.28 |
| Summer (Jun., Jul., Aug.) | 417/3,208 | 1.00 | Referent | 199/1,950 | 1.00 | Referent |
| Fall (Sep., Oct., Nov.) | 463/3,113 | 1.19 | 1.04-1.36 | 235/1,878 | 1.15 | 0.95-1.40 |
|  | **Alzheimer’s Disease** | | | | | |
|  | **Model 2^a^** | | | **Model 3^a^** | | |
|  | **n/N** | **HR** | **95% CI** | **n/N** | **HR** | **95% CI** |
| Winter (Dec., Jan., Feb.) | 367/3,147 | 1.23 | 1.06-1.44 | 184/1,888 | 1.16 | 0.93-1.44 |
| Spring (Mar., Apr., May.) | 324/3,269 | 1.14 | 0.97-1.33 | 165/2,007 | 1.03 | 0.82-1.28 |
| Summer (Jun., Jul., Aug.) | 300/3,208 | 1.00 | Referent | 149/1,950 | 1.00 | Referent |
| Fall (Sep., Oct., Nov.) | 331/3,113 | 1.19 | 1.01-1.39 | 163/1,878 | 1.03 | 0.82-1.29 |

CI, confidence interval; HR, hazard ratio; n, number of dementia cases; N, total sample size.
Analyses among participants with complete covariate data (N=12,737 for Model 2, N=7,723 for Model 3). There were no participants with missing data in covariates for Model 1.
^a^ Model 2 is adjusted for age, sex and education. Model 3 is additionally adjusted for ethnicity, body mass index, systolic blood pressure, diastolic blood pressure, smoking, history of diabetes mellitus, alcohol use, total cholesterol, high-density lipoprotein cholesterol, *APOE* ε4 genotype, history of heart failure, history of coronary heart disease, history of stroke and depressive symptoms.

# Supplementary Fig. 1 Hellmann scores by birth year

**
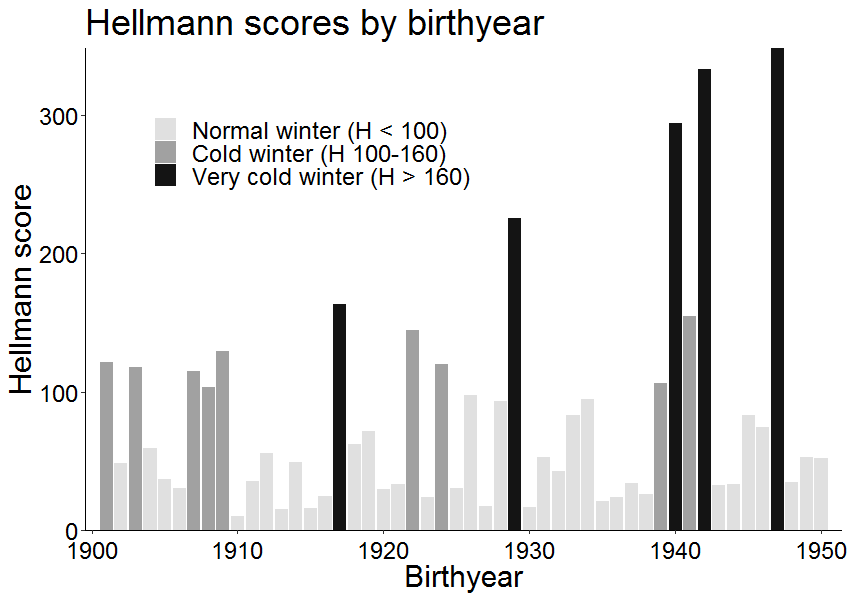
**

H, Hellmann score.
Darker bars represent colder winters.

# Supplementary Table 4 Brain imaging and season of birth

|  |  | **Coefficient (95% CI)** | | | |
| --- | --- | --- | --- | --- | --- |
|  | Intracranial volume | | Ln white matter lesions^a^ | Fractional anisotropy^b^ | Mean  diffusivity^b^ |
| Winter (Dec., Jan., Feb.) | 1.55  (-5.73, 8.82) | | -0.01  (-0.07, 0.04) | 0.00  (0.00, 0.00) | 0.00  (0.00, 0.00) |
| Spring (Mar., Apr., May.) | -1.71  (-8.85, 5.42) | | 0.02  (-0.04, 0.08) | 0.00  (0.00, 0.00) | 0.00  (0.00, 0.00) |
| Summer (Jun., Jul., Aug.) | Referent | | Referent | Referent | Referent |
| Fall (Sep., Oct., Nov.) | -1.34  (-8.62, 5.93) | | 0.01  (-0.05, 0.07) | 0.00  (0.00, 0.00) | 0.00  (0.00, 0.00) |

Coefficients from linear regression with imaging data from participants without dementia, Parkinson or stroke at the moment of scanning (N=5,237), with adjustment for age, sex and intracranial volume (except for intracranial volume itself). Coefficients are volumes (mL), ^a^natural log transformed volumes or ^b^z-scores. Reference is summer birth.

# Supplementary Fig. 2 Number of participants by year of birth


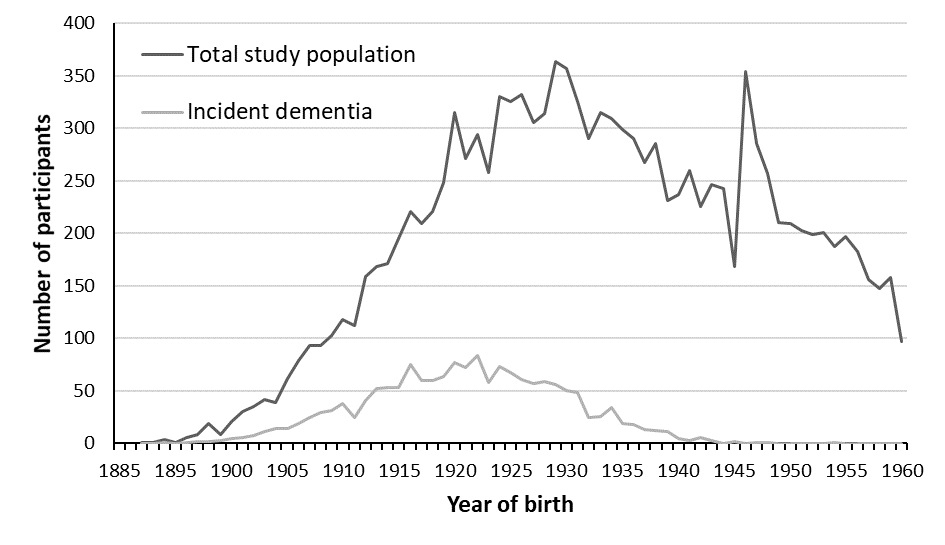


The total number of participants (dark gray) and the number of participants with incident dementia (light gray) by year of birth.
